# Diet quality among people with intellectual disabilities and borderline intellectual functioning

**DOI:** 10.1111/jar.12958

**Published:** 2021-10-26

**Authors:** David A. A. Gast, Gabriela L. C. de Wit, Amber van Hoof, Jeanne H. M. de Vries, Bert van Hemert, Robert Didden, Erik J. Giltay

**Affiliations:** ^1^ Department of Psychiatry Leiden University Medical Centre Leiden The Netherlands; ^2^ Gemiva‐SVG Group Gouda The Netherlands; ^3^ Division of Human Nutrition and Health Wageningen University & Research Wageningen The Netherlands; ^4^ Behavioural Science Institute Radboud University Nijmegen The Netherlands; ^5^ Trajectum Zwolle The Netherlands

**Keywords:** body mass index, borderline intellectual functioning, diet quality, intellectual disabilities

## Abstract

**Background:**

We sought to assess diet quality among people with intellectual disabilities or borderline intellectual functioning, living in residential facilities or receiving day care.

**Methods:**

We measured diet quality using the Dutch Healthy Diet Food Frequency Questionnaire (DHD) and compared this between participants with (*n* = 151) and controls without intellectual disabilities (*n* = 169). Potential correlates of diet quality were explored.

**Results:**

We found lower mean diet quality among people with intellectual disabilities (*M* = 80.9) compared to controls (*M* = 111.2; mean adjusted difference −28.4; 95% CI [−32.3, −24.5]; *p* < .001). Participants with borderline intellectual functioning and mild intellectual disabilities had lower diet quality and higher body mass index than individuals with severe to profound intellectual disabilities. Being female was a predictor of better diet quality.

**Conclusions:**

Overall, we found that diet quality was low in the sample of people with intellectual disabilities or borderline intellectual functioning.

## 1 BACKGROUND

Individuals with intellectual disabilities are at an increased risk of poor diet, but there is insufficient information to understand how nutritional problems are expressed in this population (Humphries et al., 2009). Obesity, diabetes and stunted growth are examples of chronic diet‐related health problems that are relatively prevalent in individuals with intellectual disabilities (Cushing et al., 2012; Ptomey & Wittenbrook, 2015). These health problems are not evenly distributed across the different severity levels among intellectual disabilities. A high prevalence of obesity (34.4%–43.9%) is found in people with mild intellectual disabilities and moderate intellectual disabilities (Hsieh et al., 2014). There is a relatively high prevalence of being underweight (10.1%) in people with severe to profound intellectual disabilities (Hsieh et al., 2014). Nutritional status among the different severity levels of intellectual disabilities needs to be systematically assessed to support effective nutritional interventions.

In this study, we focused on the dietary intake of people with intellectual disabilities or borderline intellectual functioning, which is usually assessed using food frequency questionnaires (FFQs) and food diaries (Koritsas & Iacono, 2016). Compared to the recommended daily intake, people with intellectual disabilities scored low on the dietary intake of fibres (Adolfsson et al., 2008; Bertoli et al., 2006), vegetables and fruits (Draheim et al., 2007; Hamzaid et al., 2020; Humphries et al., 2004) and poly‐unsaturated fatty acids (PUFAs; Molteno et al., 2000; Soler Marín & Graupera, 2011). In several studies, the relative proportion of saturated fats or simple carbohydrates to the total energy intake was high (Cunningham et al., 1990; McGuire et al., 2007; Robertson et al., 2000). However, the concept of diet quality goes beyond looking at the individual micro‐ or macronutrients; it aims to evaluate the entire food intake (van Lee et al., 2016; Wirt & Collins, 2009).

The relationship between diet quality and the severity of an intellectual disability has not yet been explored. Furthermore, people with borderline intellectual functioning are not often included in studies, even though they may adaptively function at the same level as people with mild intellectual disabilities (Arvidsson & Granlund, 2018). To date, no quality diet studies have been conducted among people with intellectual disabilities or borderline intellectual functioning that differentiate between the levels of intellectual disabilities. Diet studies in which diet quality was compared to a control group from the general population are also scarce.

Being overweight is linked in complex bidirectional ways to caloric intake and food choices. Sundararajan et al. (2014) found that body mass index (BMI) was inversely associated with diet quality in the general population, but there is a gap in the literature regarding the potential association between BMI and diet quality among people with intellectual disabilities.

The first aim of our study was to assess diet quality among people with intellectual disabilities or borderline intellectual functioning and to compare this assessment to the general population. The second aim of our study was to compare diet quality and BMI distribution between the people with different levels of intellectual disabilities.

## 2 METHOD

The current study was part of an overarching research project investigating the effectiveness of nutritional supplementation on aggressive behaviour among people with intellectual disabilities (clinicalTrials.gov, NCT03212092). There were two steps in the inclusion procedure. First, the inclusion criteria to participate in the informed consent procedure included having an IQ < 85 and living in a residential facility or receiving day care for at least 5 days a week. During this first step, we collected dietary and all other data used for the current analyses. Second, participants who met specific exclusion criteria regarding age, behaviour, breastfeeding, medication, morbidity or pregnancy could not proceed with the randomised controlled trial (RCT).

### 2.1 Ethical statement

The research was conducted in full accordance with the ethical principles of the World Medical Association Declaration of Helsinki. The ethical review board of the Leiden University Medical Centre (LUMC) approved the study (NL60839.058.17). All study participants or their legal representatives gave their written informed consent before the start of the data collection. Certain participants had sufficient cognitive functions to judge what participation in the study would entail, but they nevertheless had a legal representative because of their minor age or because a legal representative had been appointed by the court. In these cases, both the client and the legal representative gave written informed consent. Special versions of informed consent forms were designed to be comprehensible for people with moderate to mild intellectual disabilities.

### 2.2 Participants

All persons who gave informed consent (or for whom informed consent was given) were included in this study. We also included participants who were not included in the subsequent RCT. Participants were recruited between March 2018 and April 2020 from six intellectual disabilities service provider organisations located throughout the Netherlands. For the sake of readability, we will refer to this entire group as ‘people with intellectual disabilities’ and will only refer to ‘borderline intellectual functioning’ when it is necessary to distinguish between these groups. Two of the organisations were forensic care facilities for people with mild intellectual disabilities. The control group was drawn from of the ‘EetMeetWeet’ (EatMeasureKnow) study (www.eetmeetweet.nl). This longitudinal online study on the relationship between food and health is open to all adults who want to commit themselves to a long‐term study on this topic. For the control group, we included all participants between the ages of 12 and 40 years who applied to the ‘EetMeetWeet’ study between February 2017 and July 2017. The control group consisted of 169 participants who had a mean age of 26.4 (SD = 7.5) years.

### 2.3 Data collection

We assessed diet quality using the Dutch Healthy Diet Food Frequency Questionnaire (DHD) (www.eetscore.nl)—a questionnaire based on the 2015 Dutch food‐based guidelines for a healthy diet (Gezondheidsraad, 2015; Kromhout et al., 2016). The DHD is a short self‐report screener with 40 items and is derived from the more extensive Dutch Healthy Diet Index (Looman et al., 2017; van Lee et al., 2016). The DHD evaluates to what extent someone adheres to the Dutch Dietary Guidelines as suggested by the Health Council of the Netherlands (Gezondheidsraad, 2015). The total score ranges from 0 to 160, with higher scores indicating a better diet quality. Sixteen food groups, with a score between 0 and 10, include vegetables, fruits, whole‐wheat products, legumes, nuts, dairy, fish, tea, fats and oils, coffee, red meats, processed meats, sweetened beverages, alcohol, salt and unhealthy food products. The last group is based on the guidelines set by the Netherlands Nutrition Centre (Brink et al., 2019). For the healthy food groups, such as ‘fruits’ and ‘vegetables’, a higher intake resulted in a higher score (between 0 and 10). For unhealthy food groups, such as ‘processed meats’ or ‘sweetened beverages and fruit juices’, a higher intake resulted in a lower score. Whole grain products were based on the ratio of whole grain to refined grain, and ‘fats and oils’ were based on the ratio of saturated to unsaturated fats. For dairy, we used an optimum score of 300–450 g per day (Looman et al., 2017; see Appendix 1). In the paper questionnaires, participants provided details regarding their daily diet from the preceding month. A caregiver assisted the participants with mild intellectual disabilities when the detailed nutritional questions were too complex for them to complete independently. The caregiver completed the questionnaire as a proxy for participants with severe to profound intellectual disabilities (Table 3). We included three additional questions that determined who decided what the participants ate, how well informed the proxy was about the food habits of a participant, and who completed the DHD. The control group completed the DHD questionnaire online using an e‐form (http://www.eetscore.nl/).

Caregivers obtained the following demographic characteristics from participants: age, sex, weight (kilogramme) and height (metre). We used the case‐file data provided by the healthcare organisations to obtain participants' IQ scores. The IQ and developmental age tests were conducted by psychologists at various time points, who used the following validated tests: Bayley, Snijders‐Oomen non‐verbal intelligence test (Son‐R), Vineland Adaptive Behavior Scale (VABS), Wechsler Adult Intelligence Scale (WAIS), Wechsler Intelligence Scale for Children (WISC), Wechsler Non‐Verbal (WNV) and the Universal Non‐verbal Intelligence Test (UNIT). When a participant with severe to profound intellectual disabilities did not have a measured IQ, we estimated that participants' IQ from his or her developmental age using the WHO developmental age range as a reference (World Health Organization, 2010). The IQ cut‐off values used for the intellectual‐disabilities severity groups were <35 for profound to severe intellectual disabilities, 35–49 for moderate intellectual disabilities, 50–69 for mild intellectual disabilities and 70–85 for borderline intellectual functioning (Boat & Wu, 2015). The BMI (in kg/m^2^) was calculated and used as a potential predictor for diet quality, in addition to age and sex (Hiza et al., 2013; Temple et al., 2010).

### 2.4 Data analysis

Multivariate linear regression analysis was used to compare the DHD total and subscores between people with intellectual disabilities and controls, adjusting for age, sex and BMI. Furthermore, as potential correlates of the DHD total score, we entered the categories age, sex, BMI and IQ into a linear regression model. Using an ANCOVA, we assessed the difference in BMI between the severity groups of people with intellectual disabilities, adjusting for age and sex. Significance levels were adjusted for multiple testing based on the Benjamini–Hochberg procedure (Benjamini & Hochberg, 1995). Data were analysed using SPSS statistical software 25.0 (version 25, IBM Corp.) and the R statistical software, version 3.4.1 (R Foundation for Statistical Computing, Vienna, Austria 2016, https://www.R-project.org/).

## 3 RESULTS

Demographic characteristics of participants and controls are presented in Table 1. We included 320 participants (of whom 21.9% were women): 151 people with intellectual disabilities and 169 controls. The mean age of the group of people with intellectual disabilities was significantly higher than that of the controls. Men were overrepresented in both groups: 64.9% (people with intellectual disabilities) and 84.0% (controls). The group of people with intellectual disabilities showed a higher mean BMI than controls.

**TABLE 1:** Descriptive characteristics of participants and control subjects

	Participants with intellectual disability or borderline intellectual functioning			Controls			
	*n*	Mean (SD) or %	Range	*n*	Mean (SD) or %	Range	*p* ^a^
Male	98	64.9%		142	84.0%		
Female	53	35.1%		27	16.0%		
Age (years)	151	23.2 (7.9)	12–57	169	26.4 (7.5)	14–40	<.001
Body mass index (BMI)	149	24.9 (6.1)	14–52	168	22.7 (3.8)	16–44	<.001
IQ	142	52.6 (20.6)	10–85				
Receiving day care only	9	6.0%					
Staying in a residential facility	142	94.0%					

Abbreviation: SDAS‐11, Social Dysfunction and Aggression Scale.

a Difference between groups.

Table 2 shows the mean BMI according to intellectual disability severity group. It is noteworthy that the BMI is significantly higher in participants with mild intellectual disabilities than participants with severe and profound intellectual disabilities, *F*(3, 143) = 5.3, *p* = .002. At most locations, the food choices were made by the caregiver together with the participant (Table 3).

**TABLE 2:** BMI, mean age and proportion of females among participants

Severity of intellectual disabilities	*n*	BMI (SD)	*n*	Age in years (SD)	% female
Borderline	42	26.1 (5.9)	42	20.2 (7.4)	52.4
Mild	41	27.1 (7.8)	42	23.6 (7.9)	38.1
Moderate	22	23.4 (4.8)	22	25.3 (6.6)	27.3
Severe to profound	44	22.4 (3.9)	45	24.5 (8.5)	20.0
Total	149	24.9 (6.1)	151	23.2 (7.9)	35.1

Abbreviations: Borderline, borderline intellectual functioning; BMI, body mass index.

**TABLE 3:** Additional questions in the group of people with intellectual disabilities about the food choice and the use of proxy informants for completing the DHD (*n* = 150)

	*n*	%
Who decides what the participant eats?		
Not the caregiver nor the participant	20	13.3
Caregiver	50	33.3
Caregiver together with the participant	71	47.3
Participant only	1	0.7
Parents	8	5.3
Who completed the DHD?		
Proxy	64	42.7
Proxy together with participant	85	56.7
Participant alone	1	0.7
Does the proxy know everything the participant is eating?		
Yes, every meal including snacks	89	59.3
All meals except snacks	32	21.3
Two meals a day	20	13.3
One meal a day	8	5.3
No	1	0.7

Abbreviation: DHD, Dutch Healthy Diet Food Frequency Questionnaire.

**FIGURE 1:**
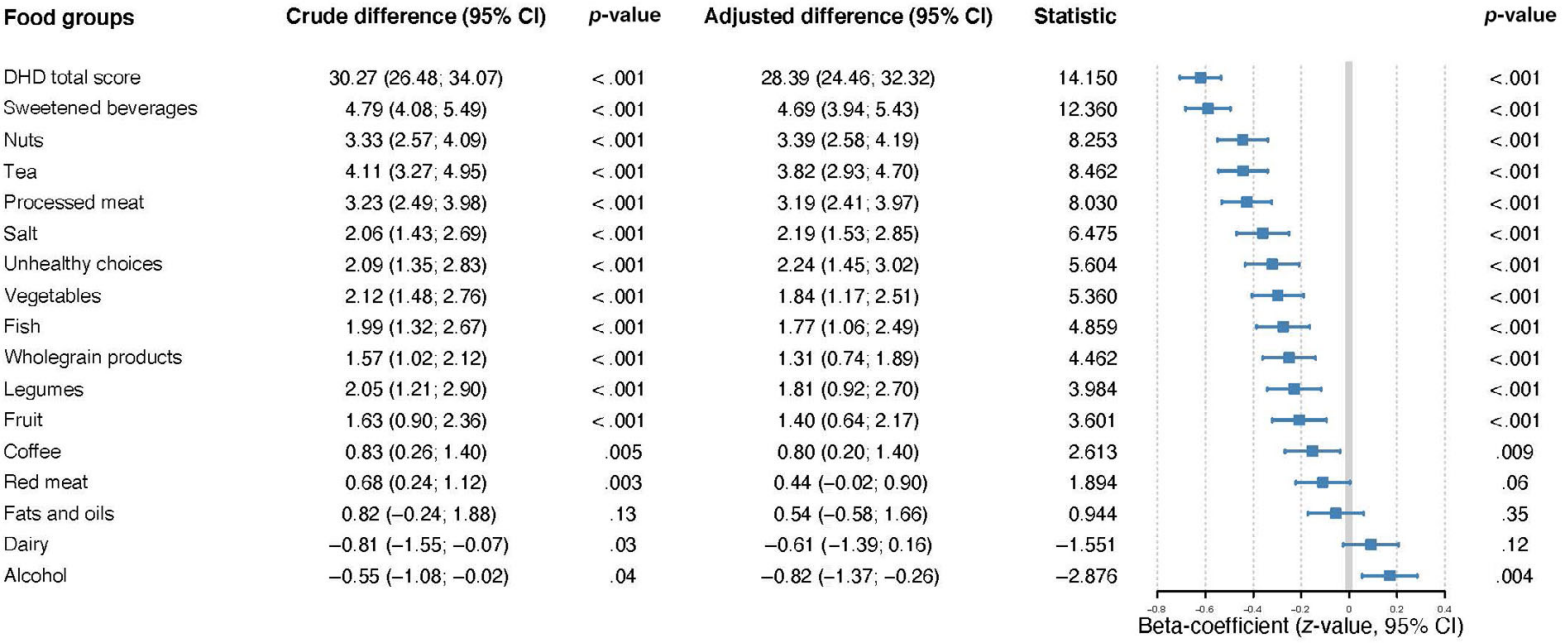
Frequency questionnaire (DHD) score of participants with intellectual disabilities and controls and their adjusted differences. DHD, Dutch Healthy Diet Food Frequency Questionnaire

Figure 1, the mean DHD total score of the participants with intellectual disabilities was 80.9 (SE ± 1.4; range: 26–18). The poorest adherence to the Dutch Dietary Guidelines was seen in the subcategories unhealthy choices (mean score 1.1), nuts (score 1.9), tea (score 2.3), processed meats (score 2.7), sweetened beverages (score 3.2) and fish (score 3.6). The best adherence was seen in the subcategories coffee (score 7.3), red meat (score 8.9) and alcohol (score 9.4). The total DHD score of participants with intellectual disabilities was on average 30 points lower compared to that of controls (80.9 vs. 111.2; *p* < .001). Furthermore, significant mean differences were observed for all subcategories except for red meat, fats and oils and dairy. All subcategory scores that differed significantly from the control group showed a lower score in the group of people with intellectual disabilities compared to the control group, except for alcohol. The largest mean differences in subcategory scores were observed for the following categories: processed meats (3.2 points lower), nuts (3.4 points lower), tea (3.8 points lower) and sweetened beverages (4.7 points lower). Significant associations persisted after adjusting for multiple testing.

### 3.1 Predictors of diet quality

**FIGURE 2:**
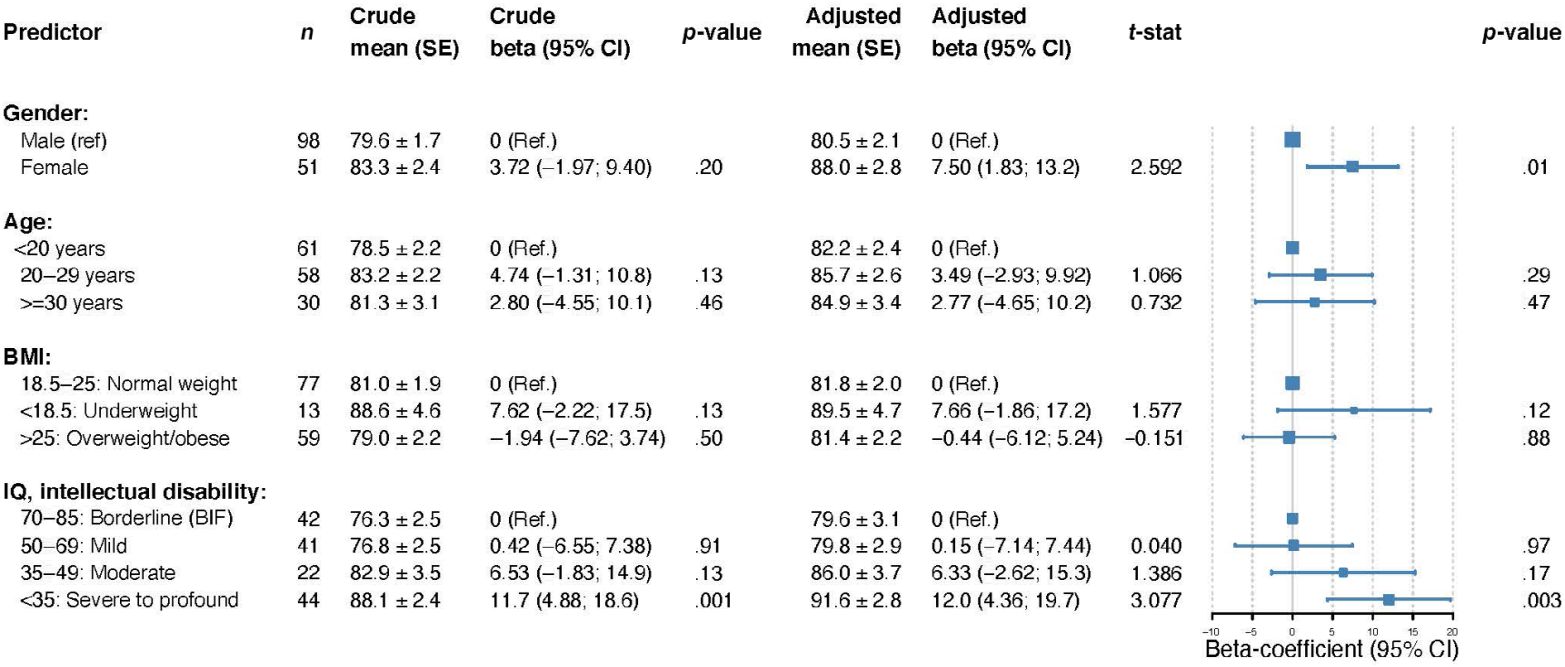
Predictors of diet quality in people with intellectual disabilities, crude and adjusted means and crude and adjusted betas with Forest plots. BIF, borderline intellectual functioning; BMI, body mass index; IQ, intelligence quotient

Figure 2 presents the analyses of the potential correlates of overall diet quality for the 151 participants with intellectual disabilities. In the multivariate analysis, women had on average a better diet quality compared to men (*p* = .01). Participants with mild intellectual disabilities and borderline intellectual functioning had a lower diet quality compared to participants with severe to profound intellectual disabilities (*p* = .007). Age and BMI groups were not significant predictors in the multivariable model.

## 4 DISCUSSION

Overall, our results showed that diet quality in participants with intellectual disabilities was lower than that of the control group. This applied to almost all food groups, with the exception of dairy products and alcohol. The general pattern was that participants with intellectual disabilities tended to over‐consume sugar, processed meats and other unhealthy food products and under‐consume omega‐3 FAs (i.e., fish and nuts). Male participants and those with mild intellectual disability and borderline intellectual functioning were at the highest risk of consuming a low‐quality diet. It is likely that a change in eating habits in these individuals will reduce the burden of disease.

The finding of an overall low‐diet quality in people with intellectual disabilities compared to the controls is consistent with previous research (Bertoli et al., 2006; Braunschweig et al., 2004; Draheim et al., 2007; Hoey et al., 2017; McGuire et al., 2007). Likewise, many studies found similar consumption patterns in the ‘unhealthy choices’ and ‘sweetened beverages’ categories (Cartwright et al., 2015; Chia‐Feng & Jin‐Ding, 2010). In our study, alcohol consumption was low in all severity categories among people with intellectual disabilities. Among people with mild intellectual disabilities; however, alcohol consumption can be similar or even higher than that found in peers of average intelligence (Didden et al., 2020). The difference in our study can be explained by the fact that many of our participants with mild intellectual disabilities had zero or restricted access to alcoholic beverages.

Although our research was not designed to study potential causes of the relatively low‐diet quality in people with intellectual disabilities, some speculations can be made. First, it is often easier and cheaper to make unhealthy food choices (Appelhans et al., 2012; Jetter & Cassady, 2006). Without proper support, people with intellectual disabilities lack the insight and money to go for the healthier choices. In previous studies among people with moderate and mild intellectual disabilities, researchers have suggested that unsupported autonomy in food choice may lead to less healthy food choices (Adolfsson et al., 2012; Bryan et al., 2000; Grammatikopoulou et al., 2008). Second, the support staff may also lack sufficient training in foods and nutrition (Humphries et al., 2004).

The low‐diet quality in people with mild intellectual disabilities is of concern. We found different intake levels of diverse food groups, which may increase the risk of weight gain and abdominal obesity (Barnes et al., 2015; Ruanpeng et al., 2017; Schlesinger et al., 2019). It is also known that the prevalence of obesity and nutrition‐related diseases in people with moderate intellectual disabilities to mild intellectual disabilities is high (Bryan et al., 2000; Hsieh et al., 2014; Ptomey & Wittenbrook, 2015; Ranjan et al., 2018). It is likely that a change in eating habits in these individuals will reduce the burden of disease.

### 4.1 Limitations

The DHD is a 40‐item screener providing a rough estimate of the diet quality. This retrospective questionnaire may be susceptible to recall bias. The method of administration of the DHD differed among the cases and controls. Participants with intellectual disabilities often needed assistance from the support staff (observer) to complete a paper questionnaire; the control group used an online version of the questionnaire as a self‐report scale. Both methods have their own risks of measurement error and bias. When filling in a self‐report scale, there may be an increased risk of participants giving socially desirable answers. When support staff helps to complete the questionnaire, some errors may be introduced because the observer is not always observing what the client is eating. Although our sample size is larger than that of previous diet quality studies in this study population, it is still small for our purposes. Moreover, participants and controls were not matched by age or gender, but we adjusted for these potential confounders in the multivariate analyses. The IQ data of the care organisations were measured using various instruments and collected at various time points, which makes a comparison of the scores less accurate. In addition, the classification of the severity level of intellectual disabilities based solely on IQ scores is outdated (Tassé et al., 2016). Since the DSM‐5, it is advised to include the level of adaptive functioning in a patient's assessment (American Psychiatric Association [APA], 2013, p. 33). Furthermore, our participants may not be representative of the whole population of people with intellectual disabilities, as they were recruited for a study on aggression and displayed higher levels of aggressive behaviour. Therefore, our findings need to be replicated in other groups of people with intellectual disabilities. Additionally, the control group may have had some self‐selection for a relatively healthy lifestyle (given the lower than average BMI) compared to the general population (RIVM, 2012).

### 4.2 Strengths

The same FFQ was used in people with intellectual disabilities and controls. Moreover, we adjusted our analysis for potential confounders, and we analysed the potential effects of different severity levels among intellectual disabilities. Additionally, the data were collected in 76 locations from four intellectual disabilities care organisations and two forensic intellectual disabilities care organisations in the Netherlands, which increased the external validity.

Even if people with moderate to mild intellectual disabilities can identify healthy food, they still need support to translate this knowledge into making healthy choices (Adolfsson et al., 2012; Kuijken et al., 2016). To sustainably increase the diet quality, more is needed apart from simply training the support staff. In a study regarding the facilitating factors for health promotion, Kuijken et al. (2019) concluded that a healthy lifestyle should be embedded in the mission of the care organisation and in the individual support plans of the clients with intellectual disabilities and should also be part of the employees' job descriptions. Diet quality among people with intellectual disabilities might be improved through a deeper integration into the entire care system.

## CONFLICT OF INTEREST

The authors declare that there is no conflict of interest.

## Supporting information


**Appendix** S1: Supporting Information

## Data Availability

The data that support the findings of this study are available from the corresponding author upon reasonable request.
