# Supplementary material for: Diet quality among people with intellectual disabilities and borderline intellectual functioning
Source: J Appl Res Intellect Disabil. 2021 Oct 26;35(2):488–94. doi: 10.1111/jar.12958 (PMC9298221; doi:10.1111/jar.12958)
Supplement: Supplementary file 1 — Appendix S1: Supporting Information [file JAR-35-488-s001.docx]

**Appendix**

Components of the Dutch dietary guidelines with the maximum and minimum score based on the DHD15-index (Looman *et al.* 2017).

|  | | | |
| --- | --- | --- | --- |
| **Component** | **Recommendations Dutch dietary guideline** | **Minimum score (=0)** | **Maximum score (=10)** |
| Vegetable | Eat at least 200 gram vegetables / day | 0 g | ≥ 200 g |
| Fruit | Eat at least 200 gram fruit / day | 0 g | ≥ 200 g |
| Wholegrains | 1. Eat at least 90 grams of brown, whole grain bread or other whole grain products per day (50%).  2. Replace refined grain products with whole grain products (50%). | 0 g  No consumption of whole grain products OR ratio of whole grain to refined grain products ≤ 0.7 | ≥ 90 g  No consumption of refined grain products OR ratio of wholegrain / refined grain products ≥ 11 |
| Legumes | Eat legumes weekly | 0 g | ≥ 10 g |
| Nuts | Eat at least 15 grams unsalted nuts / day | 0 g | ≥ 15 g |
| Dairy | Take a few servings of dairy a day, including milk and yogurt | 0 g OR ≥ 750 g | 300-450 g |
| Fish | Eat fish, preferably fatty fish, once a week | No fish consumption | Consumption of fish at least 4 times a month, of which at least 3 times fatty fish. |
| Tea | Drink three cups of tea a day | 0 ml | ≥ 450 ml |
| Fats and oils | Replace butter, hard margarine and cooking and frying fat with soft margarine, liquid baking and frying fat and vegetable oils. | No consumption of soft margarines, liquid shortening and vegetable oils OR ratio of liquid shortening / hard cooking fat ≤ 0.6 | No consumption of butter, hard margarines and hard cooking fats  OR ratio of shortening / hard cooking fat ≥ 13 |
| Coffee | Replace unfiltered with filtered coffee | Consumption of unfiltered coffee | Consumption of only filtered coffee or no coffee consumption |
| Red meat | Limit the consumption of red meat | 100 g | ≤ 45 g |
| Processed meat | Limit consumption of processed meat | ≥ 50 g | 0 g |
| Sugar containing beverages | Drink as few sugar containing beverages as possible | ≥ 250 g | 0 g |
| Alcohol | Do not drink alcohol, or at least no more than 1 glass a day | ♀ 2 glasses or more a day OR binge drinking (4 glasses or more per day)  ♂ 3 glasses or more a day OR binge drinking (6 glasses or more per day) | No alcohol, or no more than 1 glass a day |
| Salt | Eat no more than 6 grams of table salt per day | ≥ 3.8 g (sodium) | < 1.9 g (sodium) |
| Unhealthy choices* | Energy dense and nutrient poor food items not included in one of the 15 DHD components | Less than 3 unhealthy choices | 7 or more unhealthy choices |
| * “Unhealthy choices” are added to the DHD-15 components and consist of food products that contribute significantly to the total energy intake | | | |
